# Effects of β-Hydroxy β-Methylbutyric Supplementation in Combination with Conservative Non-Invasive Treatments in Athletes with Patellar Tendinopathy: A Pilot Study

**DOI:** 10.3390/ijerph19010471

**Published:** 2022-01-01

**Authors:** Ángela Sánchez-Gómez, Jose Manuel Jurado-Castro, Fernando Mata, Antonio Jesús Sánchez-Oliver, Raúl Domínguez

**Affiliations:** 1 Departamento de Enfermería Farmacología y Fisioterapia, Facultad de Medicina y Enfermería, Universidad de Córdoba, 14004 Córdoba, Spain; asgomez@uco.es; 2 Metabolism and Investigation Unit, Maimonides Biomedical Research Institute of Cordoba (IMIBIC), Reina Sofia University Hospital, University of Cordoba, 14004 Córdoba, Spain; juradox@gmail.com; 3 Centro Adscrito a la Universidad de Sevilla, Escuela Universitaria de Osuna, 41640 Osuna, Spain; 4 Centro de Estudios Avanzados en Nutrición, 14010 Córdoba, Spain; fmataor@gmail.com; 5 Departamento de Motricidad Humana y Rendimiento Deportivo, Universidad de Sevilla, 41013 Seville, Spain; rdherrera@us.es; 6 Studies Research Group in Neuromuscular Responses (GEPREN), University of Lavras, Lavras 37200-000, Brazil

**Keywords:** recovery, injury, patellar tendinopathy, rehabilitation, supplement, sport nutrition

## Abstract

The aim of the present study was to analyze the effect of conservative non-invasive treatments based on eccentric training, stretching and extracorporeal shock wave therapy (ESWT) supplemented with β-Hydroxy β-methylbutyric (HMB) or placebo (PLAC) on body composition, pain and muscular function (jump ability, muscular power and muscular strength) in athletes with patellar tendinopathy (PT). In a double-blind randomized trial, 8 athletes (4 males and 4 females) performed a physical rehabilitation for 4 weeks. They were randomly divided into two experimental groups (two males and two females in each one) that ingested HMB (HMBG) or PLAC (PLACG). In pre- and post-intervention were assessed body composition, pain, countermovement jump (CMJ), back-squat (BS) for analyzing peak power (W) (PP_PP_), load (kg) associated to PP_PP_ (PP_KG_) and mean velocity (m/s) (PP_MV_) in addition to a 5-RM leg extension tests. An interaction intervention·supplementation (*p* = 0.049; Ƞ^2^_p_ = 0.774) was observed in the height reached in the CMJ as an intervention effect in PPPP detected for the HMBG (*p* = 0.049). In addition, an enhancement in PPKG (*p* = 0.028; Ƞ^2^_p_ = 0.842) was detected in the intervention, but not in PPMV, as an increase in the intervention in the 5-RM test (*p* = 0.001; Ƞ^2^_p_ = 0.981) was observed. No changes were noted on body composition or pain (*p* > 0.05). The combination of eccentric training with stretching and ESWT increased concentric muscular power and strength after 4 weeks without changes in body lean mass or pain. In addition, HMB supplementation could enhance the power muscular performance in athletes with PT, optimizing the intervention adaptions.

## 1. Introduction

Patellar tendinopathy (PT) is one of the most common musculoskeletal pain problems associated with sports, particularly those that includes jumping activities [1], including a prevalence ~50% in high-level volleyball [2] and basketball players [3], being frequently called “jumper’s knee” [4]. PT is basically caused by tendon overload [5] and affects the insertion of the patellar tendon just under the apex of the patella [6]. PT is characterized by the increased presence of fibroblasts, vascular hyperplasia, increased amounts of proteoglycans and glycosaminoglycans, disorganized collagen, absence of inflammatory cells and prostaglandin [7]. It is accompanied by a symptomatology of pain in the anterior aspect of the knee and limits the athlete’s functional capacity and, therefore, the performance of sports actions that over-solicit the tendon [8].

Invasive strategies, such as platelet-rich plasma [9], intratissue percutaneous electrolysis (EPI), ultrasound-guided galvanic electrolysis technique (USGET) and high-volume infiltrations [10,11], have shown diverse results in PT treatment. Conservative non-invasive treatments, such as ultrasound [12] and extracorporeal shockwave therapy (ESWT) [13] and, mainly, eccentric exercise [14,15,16] and stretching [17], constitute feasible alternatives to improve injured patella in athletes. For PT rehabilitation, the decline squat is the most commonly used type of specific eccentric exercise used [5]. During eccentric contractions, the external force is greater than that being exerted by the muscle and hence lead to the muscle lengthening while tension is generated [18]. After a single session of eccentric exercise, non-adapted muscles experienced a delayed onset muscle soreness (DOMS), which produces local pain, reducing muscle functionality by decreasing maximal voluntary contraction and range of movement (ROM) [19], alters protein synthesis and degradation, and stimulates an inflammatory response [20]. However, repeated eccentric bouts (eccentric training) foster an adaption to exercise by which the muscles involved respond more efficiently to DOMS and its side effects [21]. Besides, eccentric training promotes the alignment of collagen fibers, generating more resistant fibers, stimulating the fibroblasts activity and preventing adherences during the healing stage between the tendon and the adjacent tissues [22]. The combination of eccentric training and stretching has demonstrated a higher effect in the reduction of pain in athletes with PT [23]. In addition, ESWT could generate high forces on the tendon producing analgesic benefits by the mechanical disintegration of calcium deposits and the stimulation of tissue repair [24,25] and optimize a conservative treatment in athletes with PT [26,27].

Different studies have proposed that through nutrition it is possible to optimize physical rehabilitation in sports injuries [28,29]. Specifically, certain sports supplements have been found to be effective in recovering from injuries or after long periods of immobilization. However, less attention has been paid to the supplementation effects on musculoskeletal injuries [30]. β-Hydroxy β-methylbutyric (HMB) is a leucine metabolite resulting from this essential amino acid transamination to α-ketoisocaproic acid (α-KIC), which is subsequently converted into HMB by dioxygenase [31]. HMB has been proposed as an ergogenic sports supplement for the maintenance of the nitrogen balance based on its anabolic and catabolic effects. In vitro, it has been reported that HMB enhances protein synthesis by its involvement in the mammalian target of the rapamycin (mTOR) pathway [32], partially explained by an increased activity in the muscle of the growth hormone/IGF-1 axis that improves protein synthesis via mTOR activation [33]. In addition, in myoblast cultures, the addition of HMB increases mRNA levels of markers of activated satellite cells and protein levels of muscle differentiation factors [34]. Moreover, by antagonism of the ubiquitin–proteasome pathway, HMB could diminish the rate of degradation of intracellular proteins [35] and increase the cell membrane integrity by its effect as a precursor of intracellular cholesterol [36]. Although athlethes frequently consume HMB supplements to enhance body composition (increasing lean mass and reducing fat mass), two different meta-analyses have reported a non-significant effect of HMB supplementation on body composition in athletes [37,38]. Its absence of effects on highly-trained athletes has been explained by a lower effect on trained athletes and a higher susceptibility in untrained individuals, especially during the initial stages of training when untrained subjects present higher levels of muscle damage compared with the trained population [39]. In fact, HMB supplementation has demonstrated benefits to preserve or improve muscle mass and muscular strength in older people with muscle loss [40] and function as an alternative in the treatment of sarcopenic obesity in the elderly [41]. 

To date, no study has evaluated the effect of a nutritional intervention in athletes diagnosed with PT who undergo controlled physical rehabilitation. Physical rehabilitation of PT based on eccentric training causes a high level of degradation of muscle protein, such as promoting the synthesis of muscle proteins and collagen and the possible effects of HMB supplementation when high catabolic situations exist. Therefore, the aim of the present study was to analyze the effect of 4 weeks of physical rehabilitation that combined eccentric training, stretching and ESWT, supplemented with an HMB or placebo (PLAC) supplementation on athletes diagnosed with PT on body composition, perceived pain, and muscular function (jump ability, muscular power and muscular strength).

## 2. Materials and Methods

### 2.1. Experimental Design

**Figure 1:**
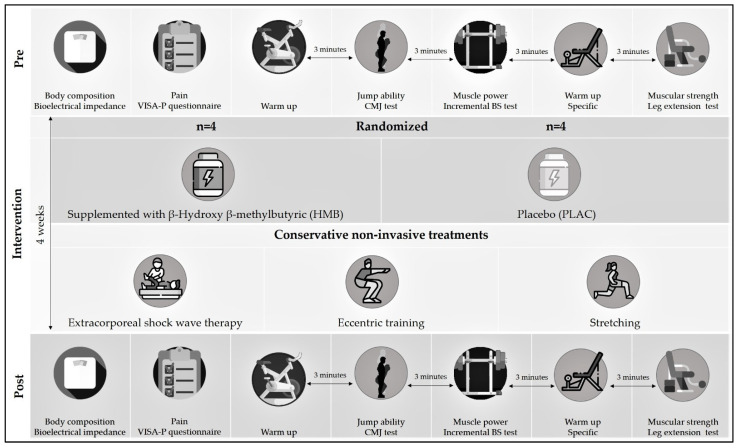
Experimental design.

The study involved an experimental double-blind randomized trial in which 8 athletes (4 males and 4 females) undergoing a 4-week patellar tendinopathy rehabilitation program of home sessions, in addition to sessions supervised and treated by a collegiate physiotherapist. Participants were randomly divided in a placebo supplementation intervention group (PLACG) or HMB supplementation intervention group (HMBG). Randomization to each experimental group (HMBG and PLACG), for ensuring a homogeneous sex-participants distribution (two males and two females in each group), was performed using Research Randomizer (www.randomizer.org). At the beginning and at the end of the study, the athletes performed an experimental session in the laboratory, – where anthropometric measurements, pain, jump ability, muscular power and strength were measured (see Figure 1). Participation in the study was voluntary and all the participants were informed of the study protocol, schedule and nature of the supplementation, the exercises, and the tests to be performed before signing an informed consent form. The study protocol adhered to the Declaration of Helsinki and was approved by an Ethics Committee (code: UI1-PI017).

BS: back squat; CMJ: countermovement jump; VISA-P: Victorian Institute of Sport Assessment-Patella.

### 2.2. Participants

All the participants were federated athletes (including basketball, volleyball, handball, and athletics) who were diagnosed with patellar tendinopathy by a sports medicine doctor. They were recruited through direct contact with the federated sports club of the city of Córdoba (Spain). In addition to being federated athletes and being diagnosed with PT by a sports medicine doctor and a physiotherapist based on the criteria of Rio et al. (2015) [42], the participants had to fulfill the following inclusion criteria: (1) aged 18–49 years; (2) not having previously undergone knee surgery or analgesic or platelet-rich plasma infiltration; (3) not having consumed any substance that could affect their hormone levels or sport performance in the previous 3 months, such as sport supplements or steroids; (4) not having any food intolerance or allergy; (5) not being a smoker; (6) not having any cardiovascular, kidney or liver disorder.

### 2.3. Supplementation 

HMB (3 g·day^−1^) in HMBG or PLAC (3 g·day^−1^ sucrose) in PLACG were ingested in three capsules 60 minutes before exercise, according to the optimal dose and timing of this supplement [43]. To ensure blinding, the supplements were provided in #1 nontransparent red capsules (Guinama S.L.U, 0044634, La Pobla de Valbona, Spain) that were prepared using a semi-automatic manual filling machine Capsunorm 2000 (Miranda de Ebro, Spain), guaranteeing food safety measures in the laboratory. In addition, to avoid any possible ergogenic effect of caffeine during the assessment sessions, a list of foods rich in caffeine (e.g., coffee, tea, tea soft drinks, cola drinks, mate, energy drinks, chocolate drinks and chocolate) was provided to all participants to avoid its consumption 24 h prior.

### 2.4. Physical Rehabilitation

The rehabilitation program was combined with an eccentric training program and extracorporeal ESWT. During the entirety of the intervention, the participants performed single-legged eccentric decline squat exercises, knee exercises (decline squat), with 3 sets of 10 repetitions being performed daily. The exercise involved standing on the painful leg, performed in a declined plane (25°) and maintaining an upright trunk while slowly squatting down in 2 sec until reaching a flexion of 90°, which guarantees the achievement point of maximum tension (60° in the patellar tendon) [44]. This exercise was complemented with a weight vest of 5 kg when the visual analogue scale (VAS) for pain assessment obtained a score of 3 points or less [45]. The eccentric training program was performed twice a day with 2 min of recovery between sets. Before and after eccentric training, the participants performed statics stretching based on research that reported gains when stretching and eccentric training was combined in patellar tendinopathy rehabilitation [23]. In addition, the participants received three sessions (with a 1-week interval) of ESWT and manual therapy in the clinic with deep manual massage of the tendon and quadriceps unloading (of both legs). ESWT treatment was applied using a Storz Duolith SD1 (Storz Medical AG, Tägerwilen, Switzerland) in sessions divided into three phases (the third was performed in quadriceps, to remove tension in the muscular belly) [46]: R-SW 15 bar, 1500 pulses, frequency 10 Hz; R-SW 2 bar, 2000 pulses, frequency 15 Hz; V-ACTOR 2 bar, 2500 pulses, frequency 25 Hz.

### 2.5. Body Composition Assessment

The anthropometrics measures were carried out with the participants barefoot and in underwear. The body height was measured with the participants erect and their head in the Francfort´s plane using a stadiometer (Seca 214, Hamburg, Germany), while the body composition was measured using a bioelectrical impedance analysis (Tanita MC-780MA, Tanita Corporation, Japan), ensuring standardized conditions for bioelectrical impedance measurement [47]. The following variables were analyzed: body mass, body mass index (BMI), body fat mass (kg), % body fat, body muscle mass (kg) and % body muscle mass. 

### 2.6. Pain Assessment

For the analysis of pain, the participants completed the Victorian Institute of Sport Assessment-Patella (VISA-P questionnaire), the only disease-specific instrument to measure PT symptoms that impact function and the ability to play sport [48].

### 2.7. Muscular Function Assessment

The jump ability was measured by the countermovement jump (CMJ) test. This test began with a warm-up, which started with 10 minutes of pedaling on the cycle ergometer (the first 4 minutes at a free intensity and the next 6 minutes at 75% of the maximum heart rate (Polar H10, Kempele, Finland)). After a 2 minutes rest, the participants performed five CMJs of increasing intensity. After a recovery of 3 minutes, the initiated test consisted of 3 CMJs, with a recovery period of 45 seconds between jumps. During the jump, an evaluator was at a distance of 1.5 m in the frontal plane to record the jump with a mobile (cell) phone (iPhone 7; Apple, Cupertino, CA, USA) at a sampling rate of 240 Hz, using the My Jump app. My Jump is an application which has indicated a good validity with a force platform (r = 0.995) [49]. The maximum height reached was registered. 

Muscle power was assessed by power produced in a back-squat (BS) exercise, in an incremental BS test, analyzing movement velocity and power during BS with a linear position transducer (v.4.1, Speed4Lift, Madrid, Spain) which has indicated a good validity with respect to the gold-standard (Trio-OptiTrack) (r = 0.95–1.00) [50]. Based on previous studies [51], the incremental BS consisted of 2 repetitions, with 2 seconds of rest between repetitions, lifting a 20 kg load at a maximum velocity of displacement for optimal muscle activation. Then, the load was increased, and the bar mean velocity measured displacement was under 0.80 m/s. When the mean velocity was above 0.80 m/s, the participants increased the load by 5 kg. The test finished after reaching the peak power (PP). The variables analyzed were the load (kg) lifted (PP_KG_), mean velocity (m/s) (PP_MV_), and peak power (W) (PP_pp_) reached in the repetition where PP was registered.

Muscular strength was assessed by a 5-repetition maximum (5-RM) test in leg extension (Selection Leg Extension, Technogym, Cesena, Italy). The specific warm-up consisted of 10 repetitions with a load corresponding to 50% of the estimated 10-RM (based on the individual assessment). After 3 minutes of recovery, the participants initiated the test based on individual assessment and there was a break of 2 minutes when the participants selected a load which could lift more or less than 5-RM [52]. 

### 2.8. Statistical Analysis

Normality distribution was contrasted with Shapiro–Wilk´s test and equality of variances with Levene´s test. For analysing the anthropometrics, perception of pain, jump ability and muscular performance variables, separate 2 × 2 independent analyses of variance for repeated measures (ANOVA-RM) were applied for each variable. Supplementation group (HMBG vs. PLACG) was introduced as an inter-subject factor, whereas intervention (PRE vs. POST) was used as an intra-subject factor. ANOVA-RM effect sizes (ES) were calculated using partial eta squared (Ƞ^2^_p_), considering small to be under 0.25, medium, the range 0.26–0.63 and large, above 0.63 [53]. A pairwise comparison was performed with the Bonferroni post hoc test. Statistical significance was set at *p* < 0.05. All the statistical tests were performed using the Statistical Package for Social Sciences (version 20.0 for Mac, SPSS™ Inc., Chicago, IL, USA).

## 3. Results

No differences were observed in the analysis of the anthropometric variables during the intervention, supplementation, or intervention supplementation (*p* > 0.05) (see Table 1).

**Figure 2:**
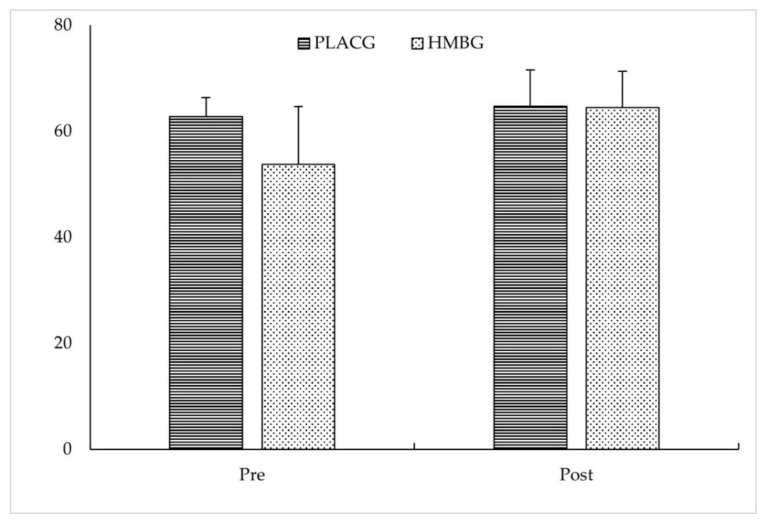
Subjective pain perceived by the VISA-P score.

In the perception of pain, neither was reported any effect for intervention (*p* = 0.080; Ƞ^2^_p_ = 0.694), supplementation (*p* = 0.251; Ƞ^2^_p_ = 0.401) nor intervention·supplementation (*p* = 0.418; Ƞ^2^_p_ = 0.226) (see Figure 2). 

HMBG: β-Hydroxy β-methylbutyric group; PLACG: placebo group; VISA-P: Victorian Institute of Sport Assessment-Patella.

The performance in the jump ability did not report differences in intervention (*p* = 0.085) or supplementation (*p* = 0.694); however, a significant effect for the interaction intervention supplementation (*p* = 0.049; Ƞ^2^_p_ = 0.774) was observed. In the incremental BS tests, an enhancement was noted in the post intervention in PP_KG_ (68.0 ± 5.1 kg vs. 62.2 ± 4.0 kg; *p* = 0.028; Ƞ^2^_p_ = 0.842), with no differences for the supplementation factor (*p* = 0.948) or the interaction of intervention supplementation (*p* = 0.335). In addition, higher values of PP_PP_ in the post-intervention were recorded (544.8 ± 40.2 W vs. 467.3 ± 40.5 W; *p* = 0.002; Ƞ^2^_p_ = 0.971), with statistical differences detected only in the HMBG (*p* = 0.049). No differences were observed for supplementation (*p* = 0.842) or the interaction intervention supplementation (*p* = 0.142) in PP_PP_. Nor were any differences noted for intervention (*p* = 0.296), supplementation (*p* = 0.268) or intervention supplementation (*p* = 0.796) in PP_MV_. As to muscular strength, a significant decrease in the weight loaded in the 5-RM test was observed (74.1 ± 2.4 kg vs. 64.4 ± 1.6 kg; *p* = 0.001; Ƞ^2^_p_ = 0.981), with differences detected as PLAG (*p* = 0.030) like in the HMBG (*p* = 0.015) (see Table 2).

## 4. Discussion

The main findings of this study were that height reached in the CMJ presented an interaction from intervention supplementation (*p* = 0.049; Ƞ^2^_p_ = 0.774) as an effect of the intervention in PP_PP_ that was only detected in the HMBG (*p* = 0.049). In addition, an enhancement in PP_KG_ (*p* = 0.028; Ƞ^2^_p_ = 0.842) and in the load weighted in the 5-RM test (*p* = 0.001; Ƞ^2^_p_ = 0.981) was detected for the intervention. In addition, an important finding is that these changes in muscular function were not accompanied by any modification in body composition variables (*p* > 0.05) and pain, measured by the VISA-P punctuation (*p* > 0.05).

Regarding the muscular strength, this study observed an enhancement in the 5-RM leg extension test after 4-weeks intervention of eccentric training combined with stretching and ESWT. This result is similar to another previous study that reported an enhancement of one repetition maximum (1-RM) on leg press after 20 weeks of eccentric training of athletes with PT [54]. Nevertheless, Romero-Rodríguez et al. (2010) found an improvement on maximal eccentric, but not concentric force after 6-weeks of eccentric training using a flywheel device in athletes with PT. These authors suggested a specific effect of eccentric training on the eccentric force [55]. However, the leg extension exercise employed in our study, the leg press exercise selected by Bahr et al. (2006), analyzes concentric strength [54]. Thus, our results confirm that, during eccentric training in athletes with PT, it could be effective to increase the concentric strength. 

In relation to muscle power, two previous studies analyzed the effect of an eccentric training program in subjects with PT through an isokinetic exercise [56,57]. In the study carried out by Biernat et al. (2014) no effect was observed in the knee flexion or extension in an isokinetic machine after 12 or 24 weeks [56], whereas Frohm et al. (2007) observed an increased power production in concentric isokinetics strength at 90 °C after 12 weeks of decline squat eccentric training [57]. In the present study, muscle power was considered to be one of the major determinants of sport performance in several sport modalities [58,59], because a common target for athletes is to apply maximum power levels to a given work load [51]. We, therefore, selected the progressive incremental BS test to determine the maximal concentric power, recognizing that the power output reflects the relation between the load and the movement velocity in the BS execution. In our research, an increase in PP_KG_ and PP_PP_, but not in PP_MV_, was observed after the intervention. These results reflect an enhancement in the power explained by an increase of the force, but not in movement velocity. The study’s results were in accordance with previous works that reported an enhancement of PP_PP_ occurring at a same PP_MV_, but with a higher PP_KG_, considering the velocity of movement at a load corresponding to peak power to be constant [51]. However, the most important finding of this study was an interaction from intervention supplementation (*p* = 0.049; Ƞ^2^_p_ = 0.774) in the height reached in the CMJ. Previously, different studies did not observe any effect on the height reached in the CMJ after 12, 20 or 24 weeks of eccentric training based on decline squat [54,56], or after 6 weeks with a flywheel device [55].

An injured tendon presents a loss of the longitudinal collagen fibers, an abruption in the collagen bundles and a relative expansion of the tendon [24,27]. The discontinuous and disorganized collagen fibers of the injured tendon reflect a loss in the functionality of this tissue [27,60]. A lower tendon stiffness and Young´s modulus, compared with healthy controls, has been observed in volleyball players with PT [61], while other studies have reported a tendon strain in subjects with PT [62]. A decrease in tendon stiffness increases the deformation to a determined force and, therefore, might be a potential cause of tendinopathy [63] and influence, over time, the muscle response (electromechanical delay) to applied force [64], which is another important risk factor for musculoskeletal injuries [65]. An effect on stiffness implies an alteration of the joint moment-angle and moment-velocity properties [66] and could explain the decreasing knee extensor in athletes with PT [67]. Thus, impairments in the muscular strength of the knee extensor muscles may be caused by a decrease in the energy-absorption capacity of the muscle-tendon complex [68]. The increased mechanic load causes an increase in the synthesis collagen [69,70]; the load caused by eccentric training could increase the content of collagen and foster the alignment of collagen fibers. This would increase the resistance of fibers and the activity of the fibroblasts and prevent adherences during the healing stage between the tendon and the adjacent tissues [22].

Eccentric training based on the 25 °C decline squat selected in our study has shown an increased knee extensor electromyography (EMG) compared to the squat performed on a flat surface [71]. The decline squat has allowed for optimizing the muscular strength of eccentric training [71] and could increase the patellar tendon strain. Moreover, it has been observed how, in the treatment of PT, interventions based on eccentric exercises seem to have shown a greater efficacy in reducing knee pain compared to an intervention based on concentric exercises [72]. In addition to the exercise selected (25 °C decline squat), we have included stretching training because an impairment flexibility in the quadriceps [73] and hamstrings has been reported [73,74] in athletes with PT. Limited hamstrings flexibility has been proposed as a factor that increases knee extensor muscles to overcome the passive resistance offered by the hamstrings [75,76], whereas flexibility deficits in knee extensors have presented strong abnormalities in the patellar tendon [77,78]. Only one study to date [23], with a methodology similar to ours, has investigated the effectiveness of eccentric training with static stretching exercises in the management of PT for 4 weeks, obtaining a greater effect of reducing pain and improving function at the end of treatment and at the follow-up when the participants performed additional static stretching. In the intervention proposed in our study, a treatment with ESWT was also carried out. ESWT has been effective at reducing muscular pain in athletes with PT by its capacity to stimulate fibroblast activity [79], type I collagen production, and tissue remodeling [80], as well as the inhibiting afferent pain-receptor function [81]. The combination of eccentric training, stretching and ESWT could potentiate the effect of this intervention on the muscular improvement in the 5-RM test and the PP in the BS tests even in the CMJ in the HMBG.

The interaction of intervention supplementation in the height reached in the CMJ and the improvement in the PP_PP_ in the HMBG, but not in the PLAG, suggests a positive effect of this supplement in combination with supervised physical therapy. Four different studies have analyzed the effect of HMB after a single eccentric training session [82,83,84]. Paddon-Jones et al. (2001) did not observe any effect during 6 days of supplementation (3.4 g/day) in a session consisting of 3 sets of 6 maximal eccentric elbow curls on an isokinetic peak torque or DOMS [83]. Nevertheless, in a study with the same protocol supplementation (3 g/day during 6 days), Knitter et al. (2000) observed a significantly lower creatine kinase (CK) and lactate dehydrogenase (LDH) response over 4 days after a 20-km run on moderately-trained athletes [82], whereas Van Someren et al. (2005) observed a lower decrease in the maximal concentric strength and the CK and DOMS peak after an eccentric session consisting of 3 sets of 10 repetitions of biceps curl unilaterally at a rate of 10 seconds for each repetition, with a load corresponding to 70% 1-RM [84]. As the CK and LDH in blood are considered commonly used markers of sarcolemma damage, the previous studies suggest that HMB supplementation over 2 weeks could decrease the muscle damage as a component within the muscle cell membrane [85] or as a precursor of intracellular cholesterol that enhances the cell membrane integrity [36]. As it is considered that eccentric exercise-induced muscle damage (EIMD) and acute losses in muscle strength, imbalances in muscle protein breakdown and protein synthesis [86], the anticatabolic effect of HMB could improve training adaptations [30]. In fact, a recent study carried out by Tsuchiya et al. (2019) observed an interaction of time·supplementation in subjects who were supplemented with HMB (3 g/day) during 2 and 4 weeks in the maximal voluntary isometric contraction torque, range of motion, DOMS and muscle stiffness after a session consisting of 6 sets of 10 maximal voluntary eccentric contraction on elbow flexors [87]. Hence, mediated by muscular stiffness, HMB could affect the acute lower muscular power production after an eccentric session. In fact, the authors speculate that a protection of the muscle membrane and muscle satellite cells may inhibit the decrease in muscle performance after eccentric exercise [87]. Given that an increase in the mechanic load causes a rise of synthesis collagen [69,70] and the positive correlation between collagen content of tendon and tendon stiffness [69,70,88], it is possible that the improvement in the recovery after each eccentric session could increase the muscular and tendon adaptation that causes an enhancement in the power lower limb observed in the CMJ and BS test in the athletes of HMBG. Also, according to the different meta-analysis performed on highly-trained athletes [37,38], HMB supplementation does not affect body composition. Our results inform that no difference in body composition is detected nor HMBG nor PLACG. Therefore, it is possible that eccentric training is effective in maintaining lean mass in athletes with PT. Regardless, it is necessary to consider that hypertrophy is not detected before 5 weeks of an eccentric training program [89] and that HMB could increase the neuromuscular adaptations during physical rehabilitation of PT. Regarding pain, different studies have observed a positive effect of eccentric training on the increase of the VISA scale above 6 [55], 12 [57,90] and 20 [54] weeks. Considering our results, where no positive effect for diminishing pain for the intervention (*p* = 0.080; Ƞ^2^_p_ = 0.694) was observed, it is possible that the duration of the intervention (4 weeks) could be too little to detect a statistical difference in pain.

The importance of this study is the novelty of using a sport supplement intervention in competitive athletes of sport modalities characterized by a high prevalence of PT caused by a similar injury mechanism. However, this study is not absent of limitations. Firstly, this study included a small sample size that included only 4 participants in each experimental group, limiting the statistical power of the results presented. Studies focused on female athletes are lower than studies focused on male athletes. Based on this, sex and exclusion criteria were not included. Nevertheless, ovulatory phases during the experimental session weren’t controlled, which could be a limitation for a possible interaction with physical performance in female participants. Thirdly, duration of the study could be a limiting factor, because four weeks could reflect neural adaption to physical training but is not sufficient for detecting structural adaptations. Finally, the absence of inclusion of a group that only uses HMB supplementation impairment to analyze the effects of HMB in PT athletes independently of doing a supervised rehabilitation program. Considering all these limitations, this study must be considered as a pilot study and future study must conducted to analyze the effect of an intervention with a higher sample size and duration

## 5. Practical Implications

Considering that PT sometimes brings the athlete’s career to an end [90], the results of this study demonstrate that the combination of different conservative treatments (stretching, eccentric training and ESWT) can optimize rehabilitation with respect to an intervention with less treatment. In addition, the positive effect of the CMJ in the HMBG suggests that it is possible to overcome jumper’s knee with a significant change in the sport performance. Consequently, through a nutritional intervention, the return period to the neuromuscular performance required for sport could be reduced.

## 6. Conclusions

The inclusion of eccentric training, selecting a decline squat (25 °C) in combination with stretching and ESWT, has reported increasing concentric muscular strength and concentric muscular power, without changes in perceived pain and body lean mass. Considering the high prevalence of PT in the athletics population and the positive results of the non-invasive treatment of the injury in just a period of 4 weeks, it is recommended to include the combination of these three treatments (eccentric training, stretching and ESWT) in the rehabilitation programs of athletes. 

The results of the present study have demonstrated that a nutritional intervention can potentiate the effectiveness of a rehabilitation of PT in athletes; thus, a supplementation with HMB (3 g·day^−1^) can enhance the power of muscular performance in athletes with PT, optimizing the adaptions of an intervention for the non-invasive treatment of the injury. Nevertheless, due to the small sample size of the present study, this study must be considered as a pilot study. In addition, future studies should analyze the possible usefulness of other sport supplements (i.e., whey protein and creatine) during the rehabilitation programs in athletes with PT.

## Figures and Tables

**Table 1:** Anthropometric variables result.

Variable	Supplementation	Intervention		*p*-Value Intervention	*p*-Value Supplementation	*p*-Value Intervention· Supplementation
		PRE	POST			
Body mass (kg)	HMBG	79.7 ± 9.3	80.3 ± 10.7	0.950	0.924	0.456
	PLACG	79.5 ± 7.3	78.8 ± 8.4			
BMI	HMBG	24.3 ± 2.7	25.1 ± 2.1	0.387	0.927	0.367
	PLACG	24.9 ± 1.9	24.7 ± 2.1			
Body fat mass (kg)	HMBG	13.9 ± 7.2	14.8 ± 6.9	0.223	0.984	0.168
	PLACG	18.2 ± 5.6	18.6 ± 5.4			
%Body fat mass	HMBG	18.2 ± 11.0	18.9 ± 10.0	0.160	0.876	0.371
	PLACG	22.8 ± 7.0	23.6 ± 7.0			
Body muscle mass (kg)	HMBG	63.3 ± 14.3	62.2 ± 13.1	0.460	0.621	0.277
	PLACG	57.8 ± 7.9	57.7 ± 7.1			
%Body muscle mass	HMBG	77.7 ± 10.6	77.1 ± 9.8	0.710	0.544	0.888
	PLACG	73.1 ± 7.0	72.8 ± 6.4			

Data presented as M ± SD. BMI: body mass index; HMBG: β-Hydroxy β-methylbutyric group; PLACG: placebo group.

**Table 2:** Performance variables in the CMJ, BS incremental test and 5-RM test.

Variable	Supplementation	Intervention		*p*-Value Intervention	*p*-Value Supplementation	*p*-Value Intervention· Supplementation
		PRE	POST			
CMJ (cm)	HMBG	38.1 ± 10.4	41.1 ± 11.7	0.850	0.694	0.049λ
	PLACG	37.0 ± 6.0	35.6 ± 4.7			
PP_KG_ (kg)	HMBG	59.4 ± 13.0	71.5 ± 17.0	0.028 *	0.948	0.335
	PLACG	65.0 ± 17.8	64.4 ± 11.6			
PP_MV_ (m·s^−1^)	HMBG	0.78 ± 0.12	0.81 ± 0.05	0.296	0.268	0.796
	PLACG	0.74 ± 0.04	0.79 ± 0.03			
PP_PP_ (W)	HMBG	455.5 ± 105.5 #	575.3 ± 138.8	0.002 *	0.842	0.142
	PLACG	479.0 ± 125.3	514.4 ± 107.0			
5-RM test (kg)	HMBG	55.0 ± 4.1 #	68.1 ± 3.1	0.001 *	0.081	0.184
	PLACG	73.8 ± 10.5 #	80.0 ± 9.4			

Data presented as M ± SD. BS: back squat; CMJ: countermovement jump; HMBG: β-Hydroxy β-methylbutyric group; PLACG: placebo group; PPKG: peak power kg lifted; PPMV: peak power mean velocity; PPPP: peak power; 5-RM: five-repetition maximum; #: significant effect of a group in the post-intervention vs pre-intervention; *: significant effect for intervention; λ: significant effect for intervention·supplementation.
